# The role of metaorganismal lipid metabolism in human health and disease

**DOI:** 10.1097/IN9.0000000000000074

**Published:** 2025-11-07

**Authors:** William J. Massey, Jonathan Mark Brown

**Affiliations:** 1 Department of Inflammation and Immunity, Cleveland Clinic, Cleveland, OH, USA; 2 Center for Microbiome and Human Health, Cleveland Clinic, Cleveland, OH, USA; 3 Department of Cancer Biology, Cleveland Clinic, Cleveland, OH, USA

**Keywords:** lipid, microbiome, metabolism

## Abstract

Most chronic diseases including coronary heart disease, obesity, diabetes, cancer, and multiple neurodegenerative diseases are driven by dysregulated lipid metabolism. In fact, many common drugs taken by millions including aspirin, statins, fibrates, and others improve health by reorganizing systemic lipid metabolism. Although we have a wealth of information on the enzymes and pathways maintaining lipid metabolic homeostasis in our human cells, there is much less known in regard to how our gut microbiome may coordinate with the host to control systemic lipid metabolism. With advances in untargeted metabolomics, there is a rapidly expanding list of gut microbe-derived lipid metabolites with unannotated function. Many of these bacterial lipids can be assimilated into host lipids and alter host lipid metabolic processes. Here, we discuss how gut microbe-derived lipids may be further metabolized by the host through metaorganismal metabolic pathways. We also discuss the untapped therapeutic potential for targeting metaorganismal lipid metabolism for the improvement of human health.

A common hallmark of chronic diseases such as obesity, type 2 diabetes mellitus, many cancers, and atherosclerotic cardiovascular disease is low-grade chronic inflammation ^[1,2]^. It is well appreciated that microbial-associated molecular patterns are sensed by host pattern recognition receptors (PRRs) to initiate innate immune responses that contribute to systemic inflammation ^[1–3]^. Less well understood is the role of “metaorganismal endocrinology”, where gut microbe-derived metabolites can engage non-PRR host receptor systems to further shape the low-grade inflammation associated with chronic disease ^[4,5]^. There are now several examples of bacterially derived small molecule metabolites that powerfully impact inflammation in human health and disease ^[4,5]^. In contrast, there is little known in regard to how the lipid products of gut microbial metabolism are sensed by the host to maintain homeostasis within the greater human metaorganism. With advances in untargeted metabolomics, there is a rapidly expanding list of gut microbe-derived lipid metabolites. However, how lipids derived from the gut microbiome can be assimilated into host lipids or potentially alter host lipid metabolic processes is unknown. The existing body of literature has thus far focused on the microbial metabolism that generates these lipids and the host sensing of bacterial lipids at the cellular level, while the uptake, distribution, and metabolism of microbe-derived lipids by the host have largely gone uninvestigated. Unfortunately, it is often overlooked that coordinated metabolism by both microbe- and host-derived lipid metabolic enzymes likely plays an important role in host receptor sensing of bacterially derived lipids. Here, we discuss how metaorganismal metabolism of lipids can powerfully shape both normal physiology and the pathophysiology of disease. We also discuss the potential for targeting metaorganismal lipid metabolism as a therapeutic approach for many common chronic diseases.

Although the field of metaorganismal lipid metabolism is still in its infancy, several key examples highlight the powerful effects that bacterial lipids can exert on the host. One of the first of these examples was discovered in the late 1990s, when bacterially derived isomers of the polyunsaturated fatty acid linoleic acid (LA, 18:2, w-6) were isolated from grilled beef ^[6–11]^. Collectively known as conjugated linoleic acids (CLAs), several bacterially derived conjugated dienoic isomers of LA were isolated ruminant bacteria and found to have potent anticancer, antiobesity, and immunomodulatory effects in rodents ^[6–11]^. Among the multiple mechanisms that have been elucidated, CLA isomers are well known to impact host oxylipin production and signaling through host peroxisome proliferator-activated receptors, a class of nuclear hormone receptors ^[6–11]^. In addition to playing key signaling roles, CLA isomers are esterified into host lipids such as triglycerides and membrane phospholipids ^[9]^, demonstrating that bacterial lipids can be intimately intertwined with lipid metabolic processes mediated by host enzymes (Figure 1A). Following the seminal work on metaorganismal CLA metabolism and signaling, several other classes of polyunsaturated fatty acid (PUFA) metabolites originating from gut bacteria have been identified ^[12–15]^. In fact, several other microbe-derived PUFA metabolites such as 10-Hydroxy-*cis*-12-octadecenoic acid, 10-oxo-*cis*-12-octadecenoic acid (KetoA), and 10-oxo-*trans*-11-octadecenoic acid (KetoC) have potent effects on host lipid metabolism and show therapeutic potential against obesity and fatty liver disease through mechanisms such as engagement of free fatty acid receptors (ie, GPR40 and GPR120), activation of Nrf2-ARE pathway, and inhibiting TGF-β signaling ^[12–15]^.

**Figure 1.**
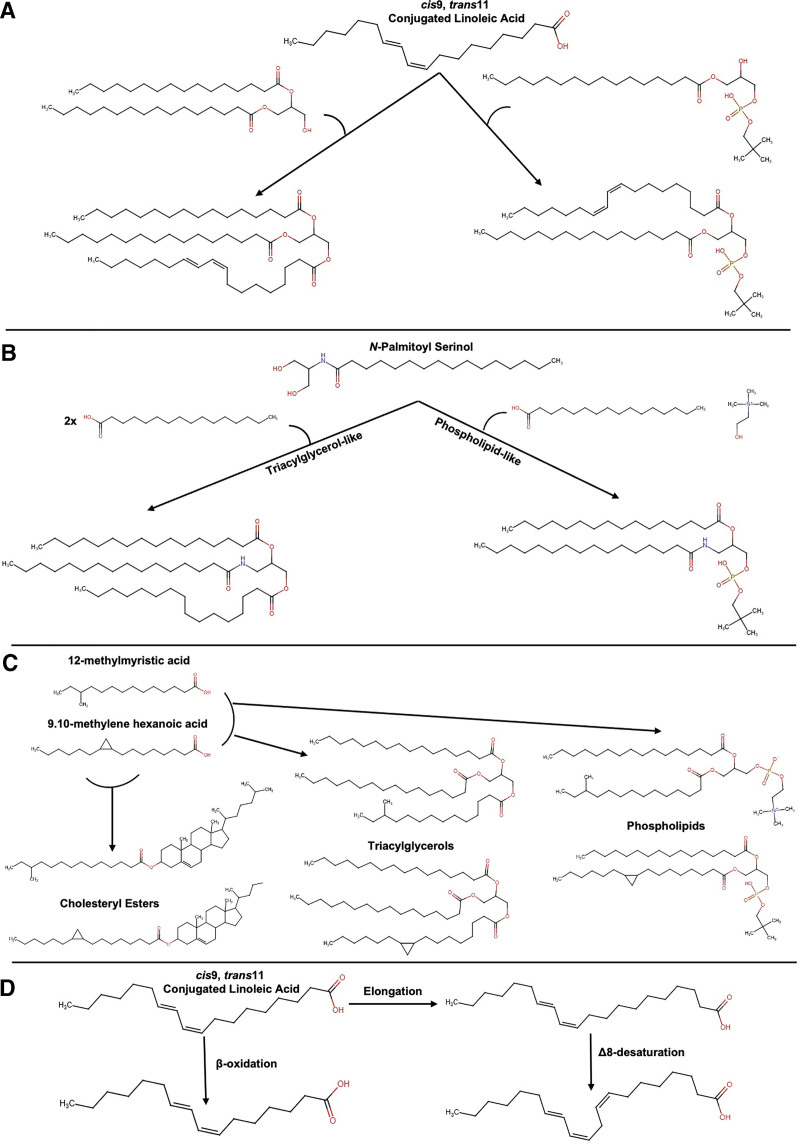
**Potential metabolic modifications of microbe-derived lipids performed by host metabolic enzymes.** (A) Conjugated linoleic acid (CLA: 18:2 Δ^9-*cis*,11-*trans*^) can be acylated into triacylglycerols (shown incorporated into *sn*-3 position) and phospholipids (shown incorporated into *sn*-1 position). (B) *N*-acyl serinols (*N*-palmitoyl serinol shown) may be acylated at their 1- and 3-hydroxy positions to form triacylglycerol-like (shown with all 16:0 acyl chains) or phospholipid-like (shown with 16:0 acyl chains and a choline head group) products. (C) Microbially modified fatty acids containing cyclopropane rings (9,10-methylene hexadecanoic acid shown) or methylation (12-methylmyristic acid) may also be incorporated into complex lipid species such as cholesterol esters, triacylglycerols, and phospholipids. (D) CLA (18:2 Δ^9-*cis*,11-*trans*^) can be β-oxidized (shown having undergone one cycle of β-oxidation forming a 16:2 Δ^7-*cis*,9-*trans*^ product) or elongated (shown as a 20:2 Δ^11-*cis*,13-*trans*^ species) and then desaturated (shown having undergone Δ8 desaturation to form a 20:3 Δ^8-*cis*,11-*cis*,13-*trans*^ product). Chemical structures were drawn in RCSB Protein Data Bank (RCSB PDB) Chemical Sketch Tool.

While research on these microbe-derived PUFA metabolites continues, a new age of functional metagenomics and other data-driven approaches have enabled the discovery of many new classes of gut microbe-derived lipid metabolites. In 2015, it was reported that lipid metabolite commendamide (*N*-acyl 3-hydroxy-palmitoyl glycine) is an agonist of the host G-protein coupled receptor (GPCR) GPR132/G2A ^[16]^, which plays key roles in monocyte chemotaxis and macrophage function contributing to atherosclerosis ^[17,18]^. Subsequently, in 2017, six classes of microbial *N*-acyl synthetases were identified that generate *N*-acyl amide lipids that mimic host *N*-acyl amide lipids such as *N*-oleoylethanolamine and agonize host receptors GPR119 and S1PR4 as well as antagonize of PTGIR and PTGER4 ^[19]^. This work was particularly important because it demonstrated a physiologically relevant role for bacterially derived *N*-acyl serinol lipids in promoting intestinal Glucagon-like peptide 1 (GLP-1) secretion to improve impact host glucose tolerance ^[19]^. Recently, our group has followed up on this initial report to show that *N*-oleoyl serinol potently impacts meal-related and circadian clock-related oscillation in host gene expression and metabolic hormone levels ^[20]^, prompting further investigation into potential incretin-like roles for bacterial *N*-acyl amides. In 2019, using crude microbial culture broth extracts, Colosimo and colleagues identified multiple microbe-derived lipid metabolites that agonized diverse orphan GPCRs, including various microbe-derived fatty acid metabolites harboring cyclopropane rings or branched chains ^[21]^. Another independent report showed that cyclopropane fatty acid metabolites originating from gut microbes can be incorporated into host membrane phospholipids ^[22]^. However, the functional consequences of cyclopropane fatty acid metabolites on host physiology or disease remain unclear. Additional studies have described the production of sphingolipids ^[23,24]^ and serine-glycine lipids ^[25,26]^ by members of the *Bacteroides* genus, which show effects on inflammation and metabolic pathways. Finally, using an elegant untargeted metabolomics approach, Mannochio-Russo and colleagues recently discovered >800 novel *N*-acyl lipids likely originating from microbial sources, and showed that circulating levels of some of these are altered by diet and are associated with diabetes, human immunodeficiency virus infection, and cognitive impairment ^[27]^. Altogether, over the past decade, there has been a flurry of report identifying novel bacterial lipids that can engage with host receptor systems (ie, GPCRs and nuclear hormone receptors) and also be substrates for host metabolic enzymes that functionally assimilate these xenolipids into the human metaorganismal lipidome.

These recent discoveries highlight the capacity of microbe-derived lipids to affect host biology through receptor-mediated signaling. In addition to receptor-mediated signaling, there are other modes through which these lipids may affect the host that deserve further exploration, such as their metabolism by host enzymes. As an example, it is possible that *N*-acyl serinol lipids may be acylated at their 1- and 3-hydroxy groups like host 2-monoacylglycerols to produce phospholipid- and triacylglycerol-like species (Figure 1B). If so, numerous questions arise as to whether these lipids would (1) have different absorption, transport, and accumulation properties than the host lipids they resemble structurally; (2) compete with the enzymatic machinery that acylates and hydrolyzes host-derived lipid species such as FAAH and PM20D1 thereby indirectly affecting host lipid metabolism; (3) serve as a reservoir that may then be hydrolyzed to produce the bioactive products that were reported to activate GPR119; and (4) may be modified through dietary manipulation. In a similar way, microbe-derived fatty acids may also be taken up by host cells and incorporated into host-derived complex lipids including phospholipids, triacylglycerols, and cholesterol esters by presently unknown mechanisms (Figure 1C). As is postulated for the *N*-acyl serinols, microbe-derived fatty acids could affect the properties of the complex lipids into which they are incorporated, compete with host-derived lipids for enzymatic activity, and act as a reservoir to activate their GPCR targets upon hydrolysis from complex lipids. Additionally, fatty acids originating from bacterial *N*-acyl lipids or other lipid metabolic products may also be subject to β-oxidation and other metabolic processing such as elongation by elongation of very long-chain fatty acids enzymes and desaturation by fatty acid desaturase or stearoyl-CoA desaturase enzymes (Figure 1D).

## 1. Conclusions

As the structural identification of microbial lipids moves forward at a record pace, the daunting task that lies ahead if functional annotation of the cellular and molecular mechanisms by which each lipid is sensed and metabolized by the host. Moving forward, it will be important to determine which host enzymes contribute to the metabolism of microbe-derived lipids across host tissues as well as to improve analytical methods enabling the robust identification and quantitation of these lipids and their derivatives in various tissue matrices. The emerging research in the area of metaorganismal lipid metabolism has the potential to have broad impact on human health and disease, given that abnormal lipid metabolism is known to be at the center of many chronic diseases within the human metaorganism.

## Author contributions

W.J.M. and J.M.B wrote, reviewed, and edited the manuscript.

## Conflict of interests

J.M.B. reports being named as coinventor on pending and issued patents held by the Cleveland Clinic relating to choline trimethylamine lyase inhibitors as therapies for cardiometabolic disease including obesity and type 2 diabetes. W.J.M. has no competing interests.

## Funding

This work was supported in part by National Institutes of Health grants R01 DK130227 (J.M.B.), R01 HL179780 (J.M.B.), P50 AA024333 (J.M.B.), and RF1 NS133812 (J.M.B.). W.J.M. is supported by a Cleveland Clinic Global Center for Pathogen and Human Health Research Postdoctoral Fellowship provided by the Infection Biology Program of the Cleveland Clinic.
